# Evaluation of Pupils’ Knowledge about Kidney Health

**DOI:** 10.3390/ijerph182312811

**Published:** 2021-12-04

**Authors:** Theodore Vassilikopoulos, Athena Kalokairinou, Georgia Kourlaba, Eirini Grapsa

**Affiliations:** 1 Nephrology Department, Aretaieio University Hospital, School of Medicine, National & Kapodistrian University of Athens, 11528 Athens, Greece; egrapsa@aretaieio.uoa.gr; 2 Faculty of Nursing, National & Kapodistrian University of Athens, 11527 Athens, Greece; athkal@nurs.uoa.gr; 3 School of Medicine, National & Kapodistrian University of Athens, 11527 Athens, Greece; kurlaba@gmail.com

**Keywords:** chronic kidney disease, childhood, school nurse, renal function, kidney health, primary school, health education

## Abstract

The purpose of this study was to investigate the level of renal function knowledge of primary school pupils in Greece. We conducted a cross-sectional study with a convenience sample of 220 pupils, coming from the 5th and 6th grades of general education schools. A questionnaire consisting of 11 questions was developed from scratch. However, based on an analysis of Cronbach’s alpha values obtained when individual questions were deleted, two questions were removed from the analysis, and only nine remained for analysis and participated in the calculation of the knowledge score. Moreover, the gender and daily habits of pupils regarding water consumption and frequency of urination were recorded. Pupils had a high percentage of correct knowledge about the number of kidneys (95.2%), whether a child may have problems with the kidneys (85.5%) and whether a person can survive with one kidney (68.5%). Low levels of knowledge were observed in the function and role of the kidneys (36.4%), as well as the part of the body where the kidneys are located (30.9%). The median (interquartile range (IQR)) total knowledge score was 6 (5–7), with no difference detected between genders (*p* = 0.135). A statistically significant difference between pupils of 5th and 6th grades was found but the difference did not seem to be clinically significant (*p* = 0.035). The present research demonstrates that pupils’ knowledge of renal function and the protection of their kidneys needs improvement.

## 1. Introduction

Chronic kidney disease is a growing worldwide public health problem [1]. It is identified by the presence of kidney damage, either structural or functional, or by a decline in glomerular filtration rate below 60 mL/min/1.73 m^2^ of body surface area for more than three months [2]. Therefore, the term chronic kidney disease defines renal dysfunction as a continuum, rather than a discrete change in renal function, both in children and adults [3]. Chronic kidney disease refers to a condition related to irreversible kidney damage that can further progress to end-stage renal disease. Chronic kidney disease is a major public health problem both in developing and developed countries [4] worldwide. While extensive epidemiological research in the adult population is available, little is known about the epidemiology of chronic kidney disease in the pediatric population. End-stage renal disease is a devastating disorder associated with excessive mortality and cardiovascular morbidity. Specific problems deriving from it occur in children, such as impaired growth and psychosocial adjustment, all of which severely impact life quality [5,6,7]. The causes of a child’s chronic kidney disease are mainly kidney and urinary tract defects, hereditary diseases and glomerulopathy [8]. Over 50% of chronic kidney diseases in children are either hereditary or congenital [9]. Worldwide epidemiologic data on the spectrum of both chronic kidney disease and acute kidney injury in children are currently limited, though increasing in scope. The prevalence of chronic kidney disease in childhood is rare—and has been variously reported at 15–74.7 per million children [4]. Such variation is likely because data on chronic kidney disease are influenced by regional and cultural factors, as well as by the methodology used to generate them [10]. Databases such as the North American Pediatric Renal Trials and Collaborative Studies [11], the United States Renal Data System [12] and the European Dialysis and Transplant Association registry [13] include data on pediatric end-stage renal disease, and a more limited range of data on chronic kidney disease. Projects such as the ItalKid [14] and Chronic Kidney Disease in Children [1] studies, the Global Burden of Disease Study 2013, as well as registries that now exist in many countries provide important information on this subject, although more is required [15].

A better understanding of chronic kidney disease epidemiology in children is essential in order to make a precise and early diagnosis, identify preventable or reversible causes of progression, predict prognosis, and aid the counseling of the children and their families [16].

Leaders in both public and school health education have long emphasized the role school health education should play in ensuring a healthy and health-literate population [17].

In 2014, the Centers for Disease Control and Prevention and the Association for Supervision and Curriculum Development (an education professional organization) collaboratively developed the Whole School, Whole Community, Whole Child framework for promoting health and academic success for school-age pupils. The Whole School, Whole Community, Whole Child framework includes 10 essential components, one of which is school-based health education [18]. As a result, teaching and learning about health has become a high priority in many schools. For example, except for violence prevention, requirements for teaching priority health topics decreased in elementary and middle schools from 2000 to 2016 [19].

The Health Promoting Schools framework is part of the World Health Organization Global School Health Initiative and has the goal of strengthening health promotion and education activities at every level from local to global [20]. The components for delivering a health-promoting school are: health policies, physical environment, social environment, community involvement, curriculum to develop health skills and the provision of health services at school [21].

It is important for pupils to obtain knowledge on the function of all human body systems, such as the urinary system and its importance in renal function. During our literature review, we found that were no corresponding studies of pupils’ knowledge on kidney health in the international or Greek literature.

School nurses have been recognized as the leaders in delivering services identified in the Healthy Child Programme from 5 to 19 years [22].

In many countries, health promotion and prevention activities in schools are part of the school nurse profession [22,23]. As a result, school nursing has evolved into a very different role from that of the traditional first aid provider [24,25].

There is a recognized relationship between health and learning, equivalent to the one between school nurse availability and student well-being and educational success [26].

School nurses are key to improving the health and wellbeing of children and young people by promoting health, providing health advice, signposting to other services, active treatment, education, family support, protection, safeguarding, service coordination and multi-agency work [22,23,24,27,28].

Unfortunately, in Greece not all public schools have nurses to support and implement health education programs for students. School nurses only offer their services in general education public schools when there is a pupil in need during school hours, and in such cases specialized nursing care is provided. For instance, a child with diabetes mellitus may receive such care.

The purpose of this study was to investigate the level of knowledge on kidney health among children attending primary school in Greece. It is hoped that this study’s findings will provide useful insights on the need to design a school education program about kidney function.

## 2. Materials and Methods

### 2.1. Study Design and Participants

This was a cross-sectional study with a convenience sample. Hence, no clustering sampling technique or sample size calculation were used. The sample consisted of 220 Hispanic pupils who attended the 5th and 6th grades of primary elementary schools of general education with good knowledge of the Greek language. They answered a self-reported questionnaire from 21 September 2020 to 24 December 2020 (4 months). Two public rural region schools in the same city were invited to participate in the study after informing their school headmasters about its purpose. After the board of teachers of each school approved the invitation, a consent form for parents was given to eligible pupils so that their parents could be informed about the purpose of the study and the process of data collection. Moreover, parents were informed that participation was voluntary and that collected data would be anonymous and confidential, following the principles of research as defined by the Declaration of Helsinki. Only pupils that returned a signed consent form participated in the study.

### 2.2. Data Collection

To collect data, a Greek questionnaire was developed which consisted of three parts: (a) demographic information (gender, school grade and age); (b) student knowledge—perception of the importance of the kidneys for good physical health; and (c) daily habits and awareness.

The questions used to assess pupils’ knowledge for renal function were:

(1) How many kidneys does the human body have?
(2) What is the main function of the kidneys in the human body?
(3) Where are the kidneys in the human body?
(4) What is the shape of the kidneys like?
(5) Is urine produced in the kidneys?
(6) Do you believe that kidney function can be controlled?
(7) What test can be used to check kidney function?
(8) Can a child have a kidney problem?
(9) Can a person live with just one kidney?
(10) Which food group helps to improve kidney function?
(11) What will happen to your body if your kidneys stop working?

For each correct answer, 1 point was assigned, while 0 points were assigned to incorrect/not sure responses.

The internal consistency (reliability) of the questionnaire was assessed by calculating the Cronbach’s alpha index, ranging from 0 to 1. Large alpha index values indicate a large coherence of the questions that make up the total knowledge score, hence high reliability. We calculated the Cronbach’s alpha values for cases where individual questions were deleted in order to identify questions that reduced the internal consistency of the questionnaire; these questions were excluded. Finally, questions 1, 2, 3, 4, 5, 6, 8, 9 and 11 were found to have moderate internal consistency (Cronbach’s alpha = 0.496). Summing the correct answers, a total score ranging from 0 to 9 was calculated, with a higher score indicating better knowledge.

Moreover, two questions regarding pupils’ daily habits and three questions of personal awareness and mobilization were asked:

(1) How much water do you drink in a day?
(2) How many times a day do you have to go to the toilet?
(3) Have you ever had a problem with your kidneys?
(4) Would you like to be informed about kidney function by your school nurse?
(5) Have you seen a poster or watched a TV advertisement about the urinary system (kidneys) recently?

### 2.3. Statistical Analysis

The collected data was statistically analyzed using the statistical package of SPSS ver. 23. The qualitative data are displayed with absolute and relative frequencies (%) as well as graphically with the use of pie charts and bar graphs. Quantitative data are presented with means, standard deviations, medians and interquartile ranges. To check the association of pupils’ answers with their demographic characteristics (gender, age), the X^2^ test of independence was used, and Fisher’s exact test was also used if deemed necessary. Differences in pupils’ knowledge scores were evaluated with the Mann–Whitney test. The level of statistical significance was set at 5% (*p* ≤ 0.05). Regarding face and content validity, the questionnaire, was completed by five schoolchildren immediately after its construction to determine whether the questions were comprehensible and clear (face validity). In addition, the same children and five health professionals who had long experience in treating kidney diseases were asked to judge the content of the questionnaire (content validity).

## 3. Results

### 3.1. Description of the Sample

A marginal majority of the sample were female (52.7%). At an identical age level, questionnaires were distributed between two grades of primary school (5th and 6th grade), that is, children aged 11 and 12 years old. In particular, 104 questionnaires were collected from the 5th grade and 116 questionnaires from the 6th grade, (47.3% and 52.7% respectively).

### 3.2. Description of the Answers Regarding Students’ Knowledge—Perception of the Importance of the Kidneys for Good Physical Health

Pupils’ answers regarding their knowledge regarding the perception of the importance of the kidneys for good physical health are presented in Table 1. Pupils had a high percentage of correct knowledge about the number of kidneys (95.2%), whether a child may have problems with the kidneys (85.5%) and whether a person can live with one kidney (68.5%). Moderate to high levels of correct knowledge were also noted regarding the fact that urine is produced by the kidneys (58.3%) and the shape of the kidneys (46.2%). Low levels of knowledge were observed in the function and role of the kidneys (36.4%), as well as wherein the body kidneys are located (30.9%).

The median (IQR) total knowledge score was 6 (5–7).

Table 2 shows that half of pupils answered the question about the consequences for kidney malfunction, with the most frequent answer being “I could die” (10.5%).

### 3.3. Pupils’ Daily Habits regarding Water Consumption and Frequency of Urination

Pupils’ answers regarding water consumption and frequency of urination are presented in Table 3. The majority of the pupils were consuming more than four glasses of water per day (73.6%) and 59.1% were urinating 2–4 times per day.

### 3.4. Description of the Answers regarding the Physical Health of the Pupils and the Willingness to Be Informed about the Kidneys

Table 3 shows the pupils’ answers about their physical health as well as their willingness to be informed about the kidneys. The vast majority of pupils had not had a problem with their kidneys (95.5%) and said that they would like to be informed about kidney function by the school nurse (95.5%). Only 7.3% had recently seen a poster or watched a TV advertisement about the kidneys.

### 3.5. The Impact of Pupils’ Gender and Grade on Their Knowledge and Daily Habits

Only 2 out of the 16 questions were statistically significantly related to gender (Table S1 in Supplementary Material). The first gender-related question (Table S1, Q-13) was concerned with the daily frequency of urination (*p* = 0.009). Girls urinated more than four times per day, which was significantly more than boys (49.1% vs. 31.7%).

The second gender-related question (Table S1, Q-15) concerned students’ interest in receiving information on kidney function from a school nurse (*p* = 0.049). The majority of responses were positive, with girls expressing greater interest (98.3%) than boys (92.3%).

No difference in total knowledge score was detected between boys and girls (*p* = 0.135, Table S2).

In Table S3, it can be seen that grade was statistically significantly correlated with pupils’ knowledge of kidney function (Q2, *p* = 0.052), their position in the body (Q3, *p* = 0.017), whether the kidneys produce urine(Q5, *p* = 0.002), on the test that can check kidney function (Q6, *p* = 0.014), whether a person can live with just one kidney (Q9, *p* = 0.001), interest in learning more about the kidneys (Q15, *p* = 0.049), and whether they had recently watched a TV advertisement (Q16, *p* = 0.004).

More specifically, older pupils (6th grade) had a statistically significantly higher percentage of correct knowledge than younger pupils (5th grade) about the location of the kidneys (37.9% vs. 23.1%), and whether a person can live with one kidney (89.5% vs. 45.1%). On the contrary, younger pupils had a statistically significantly higher percentage of correct knowledge than older pupils about the function of the kidneys (43.1% vs. 30.4%) and whether the kidneys produce urine (62.9% vs. 48.2%). In addition, older pupils expressed a greater interest in learning more about the kidneys and more often reported having recently watched a TV advertisement compared to younger pupils (98.3% vs. 92.3% and 12.1% vs. 1.9% respectively).

Regarding the total knowledge score, it was found that older pupils (6th grade) had statistically significant higher scores than younger ones (5th grade) (*p* = 0.035, Table S3), although it did not seem to be clinically significant.

## 4. Discussion

Chronic kidney disease is a major public health problem: 11–13% of the world population suffers from this disease [29]. The World Health Organization has recently added kidney and urologic disease to the mortality information tracked worldwide.

This should be a valuable source of such data over time even though the World Health Organization does not post the information by age group [30]. Data on the epidemiology of chronic kidney disease in the pediatric population are scarce. This may be explained by the lack of national registries for this disease across the world. As highlighted by Ahn et al., this explanation is very important as the number of children with chronic kidney disease is continuously increasing. These children tend to develop multiple comorbid conditions such as growth failure, developmental and neurocognitive defects, and impaired cardiovascular health [31].

Our study reveals a lack of knowledge of kidney function in childhood. In addition, the results indicate that children want to be more informed. This information provision may expand the role of school nurses, including as trainers, which in our study was proven to be highly important as there was no school nurse position in the previous years. Schools’ main responsibility is not only to promote students’ education and learning but also their awareness of health and development.

Chronic kidney disease is a global problem, and thus health education in this area during childhood may help children maintain kidney health, both at this stage and in adulthood. It is worth noting that our search of the literature in PubMed, Google Scholar and Scopus databases found no similar studies carried out in schools in Greece or in the rest of the world. We believe that the implementation of health education programs could have a positive effect on the prevention of kidney disease because during childhood the appropriate knowledge can be acquired that will help individuals to have healthy kidneys as adults. This paper describes the knowledge-perception reactions regarding the importance of the kidneys in health, as well as the ability-desire of pupils to be informed about kidney function by a specialist.

In the years since its launch, the Centers for Disease Control and Prevention, the Association for Supervision and Curriculum Development, as well as other supporting organizations have promoted the widespread adoption of the Whole School, Whole Community, Whole Child framework in schools, which includes the strengthening of health education. Distressingly, driven by controversial federal and state priorities, laws and policies associated with high-stakes testing during the preceding years [32], instruction in untested subjects (including health education) has been reduced or entirely eliminated in many schools so that learners can devote more of their attention to subjects such as reading, math, writing and science [33,34].

In Greek schools, 5th and 6th grade pupils are taught natural sciences which for example includes the study of the digestive system, the respiratory system, the circulatory system and the reproductive system [35,36]. Unfortunately, this excludes the urinary system and the kidneys’ functions.

This erosion of the education system’s commitment to raising health awareness is particularly troubling within the broader societal context. Public interest in health has become culturally pervasive as health information is now more available, more sought out and more easily accessed than ever before [37]. Technology has exponentially increased access to health misinformation and thus the sophisticated, dynamic and rapid evolution of the health sciences requires more qualified and competent guidance from all types of health education professionals [38].

School nurses need to support children in a wide range of physical and mental health problems. These problems include school bullying; chronic illnesses such as diabetes; eating disorders; obesity; grief management; mental health problems such as anxiety, depression, self-harm and post-traumatic stress disorder; and even sleep problems. In addition to assisting children with specific problems, school nurses examine children for hearing and vision problems etc. In some areas, they carry out vaccinations in schools (e.g., flu vaccination) and training in addition to providing first aid.

School nurses also play an important part in teaching children about body cognition, physical health and healthy lifestyle. In secondary schools, they educate pupils on the issues they face as teenagers, including sexual health and mental health.

Furthermore, school nurses are on the lookout for signs of student neglect and abuse and have a duty to report any concerns linked to these matters. In addition to all the above, pupils should be able to obtain further information on chronic kidney disease, which is currently a serious risk to public health, burdening social and economic sectors. Pupils should be educated on the kidneys’ functions from childhood but also the problems that a person may have if the kidneys stop functioning properly or at all. Limited public knowledge of the disease is a major barrier to the successful implementation of prevention programs. Children’s awareness of chronic kidney disease is an important determinant of the uptake of screening programs which may help to address the chronic kidney disease burden.

Besides the home, school represents the second most influential environment in a child’s life.

The school nurse is a healthcare representative in a community. An understanding of the school nurse’s role is essential to ensure coordinated care. School nurses are uniquely positioned to collaboratively assess needs in the community, collect data to formulate a plan, advocate for better health and evaluate outcomes. School nurses can expand their scope of influence by working across sectors, professions and disciplines to build a culture of health and improve health outcomes in their communities. School nurses can do this by providing leadership, advocacy, care coordination and critical thinking, and mitigating the barriers to health. The potential for school health education to improve health and save lives is significant. If we as a nation want to keep children healthy, it is important to find better ways to provide quality school health education. As is the case with most studies, the current study design is subject to certain limitations that could be addressed in future research. The first is the small sample size of schools that participated in the research. The second limitation concerns the lack of information regarding the following knowledge sources: incomplete or non-existent health education in schools, parents’ lack of knowledge or indifference in acquiring information about their children’s health, as well as inadequate information on the Internet and in health education books. The third and final limitation is the low Cronbach’s alpha coefficient.

## 5. Conclusions

In conclusion, this research illustrated the lack of Greek pupils’ knowledge on the kidneys’ functions and the protection of their kidneys. Therefore, there is a clear necessity for education and information from school nurses. However, more studies involving a greater number of children would highlight the seriousness of the problem for awareness to increase, not only nationally but also globally.

## Figures and Tables

**Table 1:** Description of the answers regarding student’s knowledge—perception of the importance of the kidneys for good physical health.

Question		Ν (%)
1. How many kidneys does the human body have? (missing: 10)	Correct	200 (95.2%)
	Incorrect	10 (4.8%)
2. What is the main function of kidneys in the human body? (missing: 6)	Correct	78 (36.4%)
	Incorrect	136 (63.6%)
3. Where are the kidneys in the human body? (missing: 0)	Correct	68 (30.9%)
	Incorrect	152 (69.1%)
4. What is the shape of the kidneys like? (missing: 8)	Correct	98 (46.2%)
	Incorrect	114 (53.8%)
5. Is urine produced in the kidneys? (missing: 4)	Correct	126 (58.3%)
	Incorrect	90 (41.7%)
6. Do you believe that kidney function can be controlled? (missing: 0)	Yes (Correct)	180 (81.8%)
	No (Incorrect)	40 (18.2%)
8. Can a child have a kidney problem? (missing: 0)	Correct	188 (85.5%)
	Incorrect	32 (14.5%)
9. Can a person live with just one kidney? (missing: 4)	Correct	148 (68.5%)
	Incorrect	68 (31.5%)
10. Which food group helps in better kidney function? (missing: 10)	Correct	132 (62.9%)
	Incorrect	78 (37.1%)
11. What will happen to your body if your kidneys stop working? (missing: 110)	Answered	110 (50.0%)
	Did not answer	110 (50.0%)

**Table 2:** Consequences for kidney malfunction.

Question	Ν (%)
11. What will happen to your body if your kidneys stop working?	
Did not answer.	110 (50.0%)
«I will not be able to breathe».	6 (2.7%)
«I will not be able to urinate/go to the toilet».	22 (10.0%)
«I will not be able to defecate».	2 (0.9%)
«The blood will be infected». «It will be very dirty».	14 (6.4%)
«The human body will not work correctly».	5 (2.3%)
«The kidneys will pick up sand and kidney stones».	4 (1.8%)
«I will not be able to walk».	4 (1.8%)
«I could die».	23 (10.5%)
«I will need a machine to clean my blood and I will need to go to the hospital every two days».	16 (7.3%)
«I will not be able to eat, drink, sleep».	4 (1.8%)
«I will be disabled».	2 (0.9%)
«I will have a urinary tract infection».	4 (1.8%)
«I need to get another kidney otherwise I will not survive».	4 (1.8%)

**Table 3:** Distribution of pupils’ answers regarding water consumption, frequency of urination, physical health and willingness to be informed about kidney function.

		N (%)
**Water Consumption**		
12. How much water do you drink a day?		
Less than 2 glasses per day		4 (1.8%)
2–4 glasses per day		54 (24.5%)
More than 4 glasses per day		162 (73.6%)
**Frequency of Urination**		
13. How many times a day do you have to go to the toilet?		
1 time per day		0 (0.0%)
2–4 times per day		130 (59.1%)
More than 4 times per day		90 (40.9%)
**Pupils’ physical health and willingness to be informed about kidney function**		
14. Have you ever had a problem with your kidneys? (missing: 0)	Yes	10 (4.5%)
	No	210 (95.5%)
15. Would you like to be informed about kidney function by your school nurse? (missing: 0)	Yes	210 (95.5%)
	No	10 (4.5%)
16. Have you seen a poster or watched a TV spot about the urinary system (kidneys) recently? (missing: 0)	Yes	16 (7.3%)
	No	204 (92.7%)

## Data Availability

The datasets generated and/or analyzed during the current study are not publicly available due to ethical restrictions, but are available from the corresponding author on reasonable request.

## Supplementary Materials

The following are available online at https://www.mdpi.com/article/10.3390/ijerph182312811/s1, Table S1; Association between gender and grade with water consumption and frequency of urination. Table S2; Association between gender and pupils’ knowledge. Table S3; Association between grade and pupils’ knowledge.
