# Supplementary material for: Evaluation of Pupils’ Knowledge about Kidney Health
Source: Int J Environ Res Public Health. 2021 Dec 4;18(23):12811. doi: 10.3390/ijerph182312811 (PMC8657303; doi:10.3390/ijerph182312811)
Supplement: Supplementary file 1 [file ijerph-18-12811-s001.zip › ijerph-1415671-supplementary.pdf]

Table S1. Association between gender and grade with water consumption and frequency of urination.

	Boys	Girls		5th Grade	6th Grade	
	**N (%)**	**N (%)**	**p-Value**			**p-Value**
**Water Consumption**			0.103			0.222
Less than 2 glasses per day	0 (0.0%)	4 (3.4%)		2 (1.9%)	2 (1.7%)	
2–4 glasses per day	29 (27.9%)	25 (21.6%)		20 (19.2%)	34 (29.3%)	
More than 4 glasses per day	75 (72.1%)	87 (75.0%)		82 (78.8%)	80 (69.0%)	
**Q13. How many times a day do you have to go to the toilet?**						
**Frequency of urination**			**0.009**			0.484
2–4 times per day	71 (68.3%)	59 (50.9%)		64 (61.5%)	66 (56.9%)	
More than 4 times per day	33 (31.7%)	57 (49.1%)		40 (38.5%)	50 (43.1%)	
**Q15. Would you like to be informed about kidney function by your school nurse?**						
**Would you like to be informed about kidney function by your school nurse?**			**0.049**			
Yes	96 (92.3%)	114 (98.3%)				
No	8 (7.7%)	2 (1.7%)				

Table S2. Association between gender and pupils' knowledge.

Question		Boys N (%)	Girls N (%)	p-Value
**1. How many kidneys does the human body have?**	Correct	92 (93.9%)	108 (96.4%)	0.386
	Incorrect/ Not Sure	6 (6.1%)	4 (3.6%)	
**2. What is the main function of the kidneys in the human body?**	Correct	40 (39.2%)	38 (33.9%)	0.422
	Incorrect/ Not Sure	62 (60.8%)	74 (66.1%)	
**3. Where are the kidneys in the human body?**	Correct	34 (32.7%)	34 (29.3%)	0.588
	Incorrect/ Not Sure	70 (67.3%)	82 (70.7%)	
**4. What is the shape of the kidneys like?**	Correct	40 (40.0%)	58 (51.8%)	0.086
	Incorrect/ Not Sure	60 (60.0%)	54 (48.2%)	
**5. Is urine produced in the kidneys?**	Correct	60 (57.7%)	66 (58.9%)	0.854
	Incorrect/ Not Sure	44 (42.3%)	46 (41.1%)	
**6. Do you believe that kidney function can be controlled?**	Correct	86 (82.7%)	94 (81.0%)	0.750
	Incorrect/ Not Sure	18 (17.3%)	22 (19.0%)	
**8. Can a child have a kidney problem?**	Correct	90 (86.5%)	98 (84.5%)	0.666
	Incorrect/ Not Sure	14 (13.5%)	18 (15.5%)	
**9. Can a person live with just one kidney?**	Correct	68 (65.4%)	80 (71.4%)	0.339
	Incorrect/ Not Sure	36 (34.6%)	32 (28.6%)	
**11. What will happen to your body if your kidneys stop working?**	Correct	50 (71.4%)	62 (79.5%)	0.254
	Incorrect/ Not Sure	20 (28.6%)	16 (20.5%)	
		Median (IQR)	Median (IQR)	
Knowledge score		6 (5–7)	6 (5–8)	0.135

Table S3. Association between grade and pupils' knowledge.

Question		5th Grade N (%)	6th Grade N (%)	p-Value
**1. How many kidneys does the human body have?**				0.422
	Correct	94 (94.0%)	106 (96.4%)	
	Incorrect/ Not Sure	6 (6.0%)	4 (3.6%)	
**2. What is the main function of the kidneys in the human body?**				**0.052**
	Correct	44 (43.1%)	34 (30.4%)	
	Incorrect/ Not Sure	58 (56.9%)	78 (69.6%)	
**3. Where are the kidneys in the human body?**				**0.017**
	Correct	24 (23.1%)	44 (37.9%)	
	Incorrect/ Not Sure	80 (76.9%)	72 (62.1%)	
**4. What is the shape of the kidneys like?**				0.143
	Correct	40 (40.8%)	58 (50.9%)	
	Incorrect/ Not Sure	58 (59.2%)	56 (49.1%)	
**5. Is urine produced in the kidneys?**				**0.002**
	Correct	72 (69.2%)	54 (48.2%)	
	Incorrect/ Not Sure	32 (30.8%)	58 (51.8%)	
**6. Do you believe that kidney function can be controlled?**				0.279
	Correct	82 (78.8%)	98 (84.5%)	
	Incorrect/ Not Sure	22 (21.2%)	18 (15.5%)	
**8. Can a child have a kidney problem?**				0.666
	Correct	90 (86.5%)	98 (84.5%)	
	Incorrect/ Not Sure	14 (13.5%)	18 (15.5%)	
**9. Can a person live with just one kidney?**				**0.001**
	Correct	46 (45.1%)	102 (89.5%)	
	Incorrect/ Not Sure	56 (54.9%)	12 (10.5%)	
**11. What will happen to your body if your kidneys stop working?**				0.562
	Correct	56 (77.8%)	56 (73.7%)	
	Incorrect/ Not Sure	16 (22.2%)	20 (26.3%)	
**15. Would you like to be informed about kidney function by your school nurse?**				**0,049**
	Correct	96 (92,3%)	114 (98,3%)	
	Incorrect/ Not Sure	8 (7,7%)	2 (1,7%)	
**16. Have you seen a poster or watched a TV spot about the**				**0,004**
	Correct	2 (1,9%)	14 (12,1%)	

**urinary system (kidneys) recently?**	Incorrect/ Not Sure	102 (98,1%)	102 (87,9%)	
		Median (IQR)	Median (IQR)	
Knowledge score		6 (5–6)	6 (5–8)	0.035
